# Long-Term Heat Stress Triggers Immune Activation and Cell Death Remodeling in the Brain of Largemouth Bass (*Micropterus salmoides*)

**DOI:** 10.3390/ani15213067

**Published:** 2025-10-22

**Authors:** Qinghui Meng, Yunye Tao, Yuhan Peng, Jie Guo, Chunfei Xun, Xiaoming Chen, Feixue Li, Huarong Huang, Fan Zhou, Jianying Li

**Affiliations:** 1 College of Life and Environmental Sciences, Hangzhou Normal University, Hangzhou 310018, China; qinghui1234@126.com (Q.M.); yunye2003@126.com (Y.T.); pengyuhan1109@163.com (Y.P.); guojie2524@163.com (J.G.); xcf_2240@163.com (C.X.); lifx@hznu.edu.cn (F.L.); jidewohhr@163.com (H.H.); 2 Zhejiang Fisheries Technical Extension Center, Hangzhou 310023, China; cxm19851219@163.com

**Keywords:** largemouth bass, brain, transcriptome, heat stress, immunity, microtubules, apoptosis, chromosome segregation

## Abstract

**Simple Summary:**

The impacts of global warming on fish physiology, particularly on the brain—a tissue highly sensitive to environmental temperature fluctuations—remain incompletely understood. In this study, we subjected largemouth bass to long-term heat stress and subsequently conducted histopathological examinations and RNA-seq analysis. Our results revealed distinct cytoarchitectural alterations in certain brain regions. Additionally, we identified 1240 differentially expressed genes. Bioinformatics analysis showed that multiple immune-related pathways were unexpectedly up-regulated, while cell death pathways were slightly activated. Concurrently, disturbances of gene sets related to microtubule dynamics and chromosome segregation were observed, leading to the inhibition of cell proliferation under heat stress. Protein–protein interaction analysis showed that 10 hub genes associated with chromosome segregation were generally inhibited, whereas ribosome biogenesis and stress response processes were enhanced in the treatment group. Collectively, our findings suggest that chronic heat stress may induce cytoarchitectural remodeling in the largemouth bass brain, accompanied by the activation of innate immunity to facilitate acclimation to environmental changes.

**Abstract:**

Heat stress typically suppresses systemic immunity in fish; however, its effects on the brain—an organ traditionally regarded as immune-privileged—remain unclear. In this study, we performed histopathological examination and RNA-seq analysis on the brains of juvenile largemouth bass (*Micropterus salmoides*) exposed to control (28 °C) and elevated (36.5 °C) water temperatures for 8 weeks. Histological analysis revealed distinct cytoarchitectural and pathological changes in specific brain regions. RNA-seq analysis identified a total of 1240 differentially expressed genes, with 22 heat shock protein genes notably showing significant up-regulation. The immune system-associated genes emerged as the most prominently affected category. Gene set enrichment analysis (GSEA) based on Kyoto Encyclopedia of Genes and Genomes (KEGG) pathway annotations revealed that up-regulated genes were enriched in immunity-related pathways, including the NOD-like receptor (NLR) signaling pathway, Toll-like receptor (TLR) signaling pathway, and cytosolic DNA-sensing pathway. Additionally, the levels of apoptosis and necroptosis were moderately increased. GSEA based on Gene Ontology (GO) terms indicated that down-regulated genes were primarily associated with cell division. Protein–protein interaction (PPI) and clustering analysis identified 41 core genes in the top three clusters, encompassing those related to nuclear chromosome segregation, ribosome biogenesis, and stress response. The inhibition of genes involved in nuclear chromosome segregation may disrupt cellular homeostasis by significantly impairing microtubule dynamics. In contrast, genes associated with ribosome biogenesis and stress response were up-regulated, which could counteract the adverse effects caused by long-term heat stress. We propose that brain-specific immune activation, particularly via the NLR and TLR signaling pathways, acts as a compensatory strategy to counterbalance heat-induced cell death, thereby revealing a novel neuro-immune adaptation axis.

## 1. Introduction

Water temperature stands as one of the most critical environmental factors governing the survival and growth of fish [1]. As ectothermic organisms, fish exhibit high sensitivity to fluctuations in water temperature, which can directly modulate their physiological processes, including metabolism, reproduction, and immune function [2]. Under optimal temperature conditions, both innate and adaptive immune systems operate efficiently, providing robust protection against infections and inflammation. Conversely, temperature deviations beyond the optimal range often lead to immunosuppression in teleost [3]. While short-term thermal stress may transiently activate certain immune responses, prolonged exposure generally has detrimental effects, prompting compensatory acclimatization [3,4,5,6]. The severity of these impacts depends not only on duration but also on the intensity and regime of the heat stress. Notably, pre-acclimation to gradually increasing temperatures has been shown to mitigate severe damage during subsequent acute thermal challenges [7,8]. Global warming is currently impacting animal populations worldwide through chronic temperature elevations and the increased frequency of extreme climatic events, which can disrupt teleost immune activity and exacerbate the occurrence of infectious diseases in aquatic ecosystems [3,9,10]. While the immunosuppressive effects of heat stress on peripheral organs (e.g., spleen, liver) have been widely reported [11,12,13], the brain—an immune-privileged site characterized by unique neuroendocrine-immune crosstalk—may employ distinct regulatory strategies [14,15]. Consequently, the impact of heat stress on brain-mediated neuroendocrine–immune interactions in fish has attracted growing research interest [1,5,7,16,17].

Largemouth bass (LMB, *Micropterus salmoides*) is a widely distributed freshwater fish species with significant ecological and economic importance, whose optimal growth temperature ranges from 26 °C to 29 °C [18]. During summer, LMBs face significant thermal stress in regions like South China, where temperatures can exceed 40 °C in July and August, particularly when they are in their juvenile stage [19]. Populations of LMB exhibit a marked increase in growth rate when inhabiting water at a supra-optimum temperature (30 °C) [20], but fail to achieve significant weight gain at 35 °C [21]. Temperatures exceeding 33 °C can cause histological damage in LMB, including endoplasmic reticulum (ER) stress, cell apoptosis, and perturbations in glucose and lipid metabolism in the liver [22,23,24]. Additionally, heat exposure induces oxidative stress, reduces the expression of immune-related genes, and increases disease susceptibility in this species [11,19]. With the completion of LMB genome sequencing [25,26], an increasing number of studies have focused on transcriptomic changes in response to thermal stress [12,23,27,28,29,30]. Nevertheless, significant gaps remain in our understanding of the molecular pathways involved in these responses, particularly in the brain—a critical organ for regulating stress and maintaining homeostasis.

The fish brain, as the central hub of the neuroendocrine system, is highly sensitive to environmental temperature changes and may even act as a determinant of thermal tolerance in fish [31]. It primarily regulates heat stress responses and stress adaptation through the hypothalamic–pituitary–interrenal (HPI) axis and the brain–sympathetic–chromaffin cell (BSC) axis [6,10,32]. Moreover, heat stress can trigger molecular regulatory mechanisms that alter gene expression in the fish brain to maintain internal homeostasis. For instance, Topal et al. [1] found that acute stress induced by rising water temperatures upregulated the expression of apoptotic genes, heat shock genes, and ER stress genes in the brains of rainbow trout. Acute thermal perturbations have also been shown to induce expression changes in proteins involved in processes such as protein synthesis, folding, degradation, and cytoskeleton organization in the brain of zebrafish [5]. In Chinese tongue sole, acute heat stress leads to differential expression of genes in the brain related to cortisol synthesis and secretion, neuroactive ligand–receptor interactions, and the JAK/STAT signaling pathway [16]. Furthermore, the expression of heat shock proteins (HSPs), specifically HSP70, in the brain is significantly upregulated following short- and long-term warm acclimation, suggesting its role in determining thermal tolerance in fish [33]. In grass carp, long-term heat stress can result in significant changes in the expression of genes in the brain associated with metabolic and immune pathways, including antigen processing and presentation, steroid biosynthesis, and the p53 signaling pathway [17]. Despite numerous reports on the effects of chronic heat stress on various fish tissues, such as the liver [24,34] and kidney [35], relatively little attention has been paid to the brain.

To simulate the summer conditions experienced by juvenile LMB and investigate the effects of heat stress on their central nervous system (CNS), we cultured juvenile LMB at high temperatures for eight weeks. Subsequent histological and transcriptomic analyses were conducted on brain tissues. Bioinformatics approaches included Gene Ontology (GO), Kyoto Encyclopedia of Genes and Genomes (KEGG), gene set enrichment analysis (GSEA), and protein–protein interaction (PPI) network analysis. This study aims to identify novel neuroimmune regulatory and adaptive mechanisms in response to heat stress, as well as to pinpoint key genes and pathways involved in thermal adaptation. The findings are expected to provide targets for developing LMB strains with improved heat tolerance through selective breeding [36].

## 2. Materials and Methods

### 2.1. Animal Domestication and Sampling

The breeding strain of LMB “Zhelu No.1” was domesticated at the Zhejiang Fisheries Technical Extension Center in Hangzhou, China. The fish were subjected to regular quarantine to prevent pathogen spread. Four hundred healthy juvenile LMB (8.63 ± 1.14 g) were evenly divided and randomly stocked into four tanks (each containing 1000 L of water), with each tank equipped with independent temperature control and aeration apparatus. Prior to the experiment, all tanks were thoroughly disinfected and then randomly assigned to two control groups and two treatment groups. The fish were introduced into the tanks one week before the experiment initiation to acclimate to the new environment and recover from handling stress. The fish were fasted for one day before the experiment started. Thereafter they were fed a commercial diet (Lianxing Feed Technology Co., Ltd., Jiaxing, China) to satiation twice daily. The amount of feed was controlled to ensure it was completely consumed within half an hour, and any uneaten pellets were removed immediately after each feeding session.

The fish were kept indoors with an air conditioner set at 28 °C. For the temperature treatment, the water temperature for the treatment group was gradually increased starting from 28 °C at a rate of 1 °C every 12 h until reaching 36.5 °C. A heating rod (JIAPU, Jiangsu Huaerwei Technology Group Co., Ltd., Huai’an, China) with an accuracy of 0.1 °C was used to adjust the temperature. Thereafter, the fish were maintained at 28 ± 0.5 °C (control group) and 36.5 ± 0.5 °C (treatment group) for 8 weeks. Water temperature in each tank was monitored twice daily using digital thermometers. Throughout the experiment, dissolved oxygen levels in the water were maintained above 6.5 mg/L. Water quality parameters were tested three times a week, with ammonia nitrogen kept below 0.5 mg/L and nitrite below 0.05 mg/L. Each week, 1/6 to 1/5 of the water in each tank was replaced. The fish were monitored at least twice daily, and any moribund or dead individuals were promptly removed and recorded.

At the end of the trial, the survival rate was calculated as the number of surviving fish divided by the initial total number of fish in each group. Fish from each tank were randomly selected and anesthetized with 250 mg/L MS-222 (Sigma-Aldrich, St. Louis, MO, USA) in a water bath before dissection. Brains from three to four fish were combined to form a mixed sample. Three such samples per group were prepared for subsequent RNA-seq and qPCR analyses. After washing with ice-cold PBS, the whole brain tissues were immediately frozen in liquid nitrogen and then stored at −80 °C until use.

### 2.2. Histology Analysis

Freshly collected samples were washed with PBS and subsequently fixed overnight in 4% paraformaldehyde (PFA). Following fixation, the tissues were dehydrated through a graded ethanol series (30%, 50%, 70%, 90%, 95%, and 100%) and then embedded in paraffin. Sections of 7 μm thickness were prepared from the paraffin-embedded tissue blocks using a Leica RM2235 microtome (Leica Microsystems, Nussloch, Germany). Hematoxylin and eosin (H&E) staining was carried out according to standard protocols. The stained sections were imaged with a Nikon Eclipse 80i microscope equipped with a DS-Fi3 camera (Nikon Corporation, Tokyo, Japan) and controlled using NIS-Elements F software (v4.60.00). Three biological replicates were prepared for each experimental group.

### 2.3. RNA Preparation, Library Construction, and Sequencing

Total RNA was extracted from the mixed brain samples using TRIzol reagent (Invitrogen, Carlsbad, CA, USA) according to the manufacturer’s instructions. Three such biological replicates per group were used for library construction and sequencing. The RNA quantity and integrity were assessed with a Bioanalyzer 2100 system (Agilent Technologies, Santa Clara, CA, USA) to meet experimental standards. Library construction was performed using a MGIEasy RNA library preparation kit (MGI, Shenzhen, China). In brief, mRNA was enriched using oligo-dT magnetic beads, then fragmented and converted into double-stranded cDNA. The purified cDNA fragments underwent end-repair, tailing, adaptor ligation, and PCR amplification.

Sequencing was conducted on a DNA nanoball (DNB) technology platform. Single-stranded PCR products were created through denaturation and then circularized. DNBs were formed from these circularized DNA molecules via rolling circle amplification. High-intensity DNA nanochip technology was used to prepare DNB nanoarrays, which were sequenced on the DNBSEQ-T7 platform (BGI, Shenzhen, China) with a paired-end sequencing length of 150 bp (PE150).

### 2.4. RNA-Seq Data Processing and Differential Expression Analysis

The raw data were filtered using SOAPnuke software (v1.6.5) to remove adapter sequences and low-quality reads. The clean reads were then mapped to the LMB reference genome (NCBI RefSeq: GCF_014851395.1_ASM1485139v1) using HISAT2 software (v2.2.1) and aligned to the reference gene with Bowtie2 (v2.4.5). Cufflinks (v2.2.1) was used to predict novel protein-coding transcripts. Principal Component Analysis (PCA) was conducted using the prcomp function from the R statistical environment and visualized with the ggplot2 (v3.4.0) package.

The Fragments per Kilobase of Transcript per Million fragments mapped (FPKM) method was used to measure gene expression levels. Differentially expressed genes (DEGs) were identified using DESeq2 (version 1.42.2) with the following thresholds: an adjusted *p*-value (False Discovery Rate, FDR) < 0.05 and an absolute log_2_ fold change (|log_2_FC|) > 0.58. The pheatmap function was used to create a heatmap of differential gene clusters.

### 2.5. Real-Time Quantitative PCR Analysis

Total RNA extracted for library construction and sequencing was reverse-transcribed for qPCR analysis. Single-stranded cDNA was synthesized from 1 μg of total RNA using the PrimeScript RT Reagent Kit with gDNA Eraser (Takara, Dalian, China), following the manufacturer’s instructions to ensure genomic DNA removal prior to reverse transcription. The cDNA was then amplified using the StepOnePlus™ Real-Time PCR System (Applied Biosystems, Carlsbad, CA, USA) with UltraSYBR Mixture (High ROX) (Cowin Biotech, Taizhou, China) and gene-specific primers (Supplementary Table S1). Gene-specific primers were designed using Oligo 7 software; the specificity of each primer pair was subsequently confirmed using the online Primer-BLAST tool. The thermal cycling conditions included an initial denaturation at 95 °C for 10 min, followed by 40 cycles of 95 °C for 15 s and 60 °C for 1 min. Melt curve analysis was performed with one cycle at 95 °C for 15 s, 60 °C for 1 min, and a gradual increase to 95 °C at 0.3 °C/s, maintained for 15 s. The 18S rRNA gene served as an internal control, with its stability validated by qPCR across various tissues of LMB. Three biological replicates were used for the qPCR analysis. Relative gene expression levels were determined using the 2^−ΔΔCT^ method [37].

### 2.6. Functional Enrichment Analysis

Functional classification of the differential genes was conducted using GO and KEGG annotations. GO enrichment analysis was performed with the TermFinder (v0.86) package, and KEGG pathway enrichment analysis was done using the phyper function for hypergeometric testing. Significant enrichment was set at FDR ≤ 0.05. Pathway graphics were created using the SRplot web server (http://www.bioinformatics.com.cn/SRplot (accessed on 19 October 2025)) [38]. To further summarize the gene sets’ functions in the transcriptomic data, GSEA (v4.3.2) was applied to explore underlying biological processes and signaling pathways [39].

### 2.7. Protein–Protein Interaction (PPI) Analysis and Hub Genes Screening

The orthologs of the DEGs were identified using the Blastx algorithm in the local NCBI BLAST+ program (version 2.14.0) against the zebrafish RefSeq protein sequence database. The best matches with an *E*-value < 1 × 10^−5^ were selected. The corresponding gene symbols were then submitted to the STRING online tool (version 12.0) to construct an interaction network with a minimum interaction score of 0.700. The PPI network was further analyzed using Cytoscape software (version 3.10.1). The MCODE plug-in in Cytoscape was used to conduct modular analysis on the PPI network with default settings (degree cutoff = 2, node score cutoff = 0.2, k-core = 2, and max depth = 100) [40]. Additionally, the CytoHubba plug-in was utilized to identify hub genes through the Maximal Clique Centrality (MCC) topological analysis method [41].

## 3. Results

### 3.1. Animal Rearing and Histopathological Analysis

**Figure 1:**
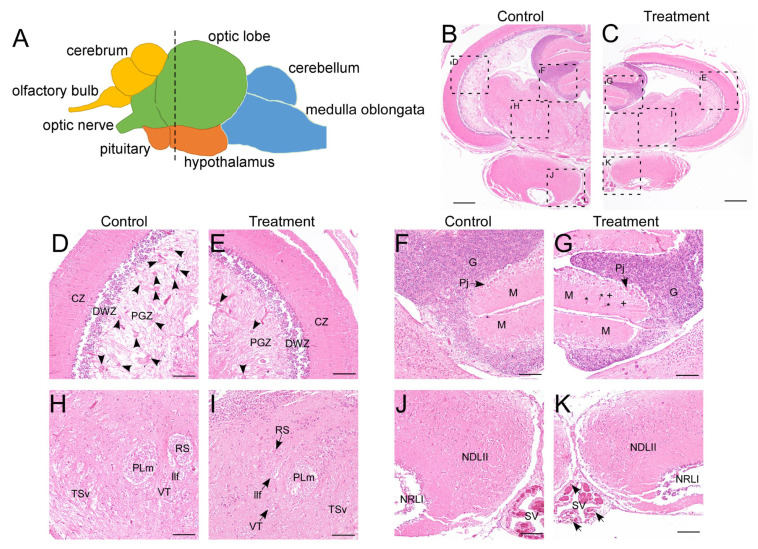
Histopathological analysis of the brain. (**A**) schematic of the LMB brain (lateral view). Different brain regions are marked with colors: yellow for the telencephalon, green for the mesencephalon, orange for the diencephalon, and blue for the rhombencephalon. The dotted line marks the location of the transverse sections in the entire brain; (**B**–**K**) H&E staining of the brain in control and treatment groups. (**D**) and (**E**), (**F**) and (**G**), (**H**) and (**I**), (**J**) and (**K**) are enlarged views of different regions in (**B**) and (**C**), showing the optic tectum, cerebellum, ventral tegmentum, and hypothalamus, respectively. CZ, central zone of the optic tectum; DWZ, deep white zone of the optic tectum; G, granular layer of the cerebellum; llf, lateral longitudinal fascicle; M, molecular layer of the cerebellum; NDLII, lateral part of the diffuse nucleus of the inferior lobe; NRLI, lateral part of the nucleus of the lateral recess; PGZ, periventricular gray zone of the optic tectum; Pj, purkinje cells; PLm, medial part of perilemniscular nucleus; RS, superior reticular nucleus; SV, saccus vasculosus; TSv, ventral part of semicircular torus; VT, ventral tegmental nucleus. Arrow heads in (**D**,**E**) indicate the radial glial cell clusters in PGZ. Asterisks indicate blood cells, and plus signs denote suspected granule cells in (**G**). Arrows in (**K**) point to melanomacrophages. Scale bars: 400 μm in (**B**,**C**), 100 μm in (**D**–**K**). The cytoarchitecture of the LMB brain was referenced according to Ref. [42].

Following 8 weeks of thermal exposure, significant physiological impacts were observed. The treatment group exhibited markedly reduced survival rates (<60% vs. >95% in controls) and substantially lower body weights (24.79 ± 8.77 g vs. 61.32 ± 10.52 g in controls). In LMB, the brain is anatomically divided into four primary parts: the telencephalon, mesencephalon, diencephalon, and rhombencephalon, which can be further subdivided into nine distinct regions (Figure 1A) [42]. Notably, the treatment group exhibited visibly smaller brain size, indicative of growth suppression (Figure 1B,C). Histopathological observations revealed distinct cytoarchitectural alterations in several brain regions (Figure 1B–K). Specifically, in the control groups, numerous cell clusters were distributed within the inner layer of the periventricular gray zone (PGZ) of the optic tectum—the stratum periventriculae, which is composed of undifferentiated neural precursor cells or glial cells. In contrast, the treatment groups showed a notable reduction in such cell clusters, accompanied by a visible increase in neurofilaments (Figure 1D,E). In the cerebellum, the presence of blood cells and suspected granule cells within the molecular layer suggested disruption of neural tissue integrity (Figure 1F,G). Furthermore, structural boundaries in the ventral tegmentum—such as those delimiting the superior reticular nucleus and the ventral tegmental nucleus—appeared blurred in treated fish (Figure 1H,I). Although the hypothalamus showed no overt morphological changes, an increased accumulation of brownish-yellow melanomacrophages was observed along the margins of the saccus vasculosus (Figure 1J,K). Collectively, these findings indicate that prolonged high-temperature exposure induces severe and widespread neural damage in the LMB brain.

### 3.2. Transcriptome Data Processing and Identification of Differentially Expressed Genes

**Figure 2:**
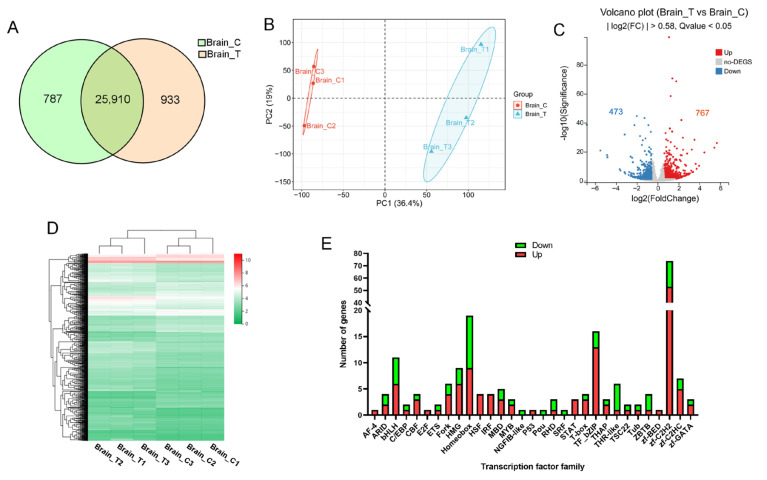
RNA-seq analysis of the brain. (**A**) Venn diagram of genes identified in control (Brain_C) and treatment (Brain_T) groups; (**B**) PCA of gene expression in control and treatment groups; (**C**) volcano plot of DEGs. Red dots indicate significantly upregulated (FDR < 0.05, FC > 1.5) genes, while blue dots represent those that are downregulated (FDR < 0.05, FC < 0.67); (**D**) hierarchical clustering analysis of gene expression across samples. The color represents the FPKM values of each gene; (**E**) statistical analysis of up-regulated (red) and down-regulated (green) genes in various transcription factor families.

RNA sequencing generated 266 million raw reads, with 253 million high-quality reads retained after filtering (Q20 > 96%, Q30 > 90%; Supplementary Table S2). These data indicate the high quality of library construction and sequencing of experimental samples. Alignment rates exceeded 92% (unique mapping > 61%) against the reference genome. More than 73% of genes mapped uniquely to the known gene set. We identified 26,697 genes in the control group and 26,843 in the treatment group, with 25,910 common genes (Figure 2A). PCA demonstrated clear separation between the control and treatment groups along PC1 (36.4% variance), indicating substantial transcriptomic reprogramming due to high temperature exposure (Figure 2B). Differential expression analysis identified 1240 significant DEGs (|log_2_FC| > 0.58, FDR < 0.05), comprising 767 upregulated and 473 downregulated genes (Figure 2C). Hierarchical clustering confirmed distinct gene expression between the control and treatment groups (Figure 2D, Supplementary Table S3). Transcription factor analysis revealed preferential upregulation of regulatory elements in stressed specimens (Figure 2E). To verify the reliability of the DEGs identified through RNA-Seq analysis, we conducted qPCR on randomly selected genes. The expression trends observed via qPCR for all tested genes were consistent with the RNA-seq data (Supplementary Figure S1).

### 3.3. GO and KEGG Enrichment Analysis

**Figure 3:**
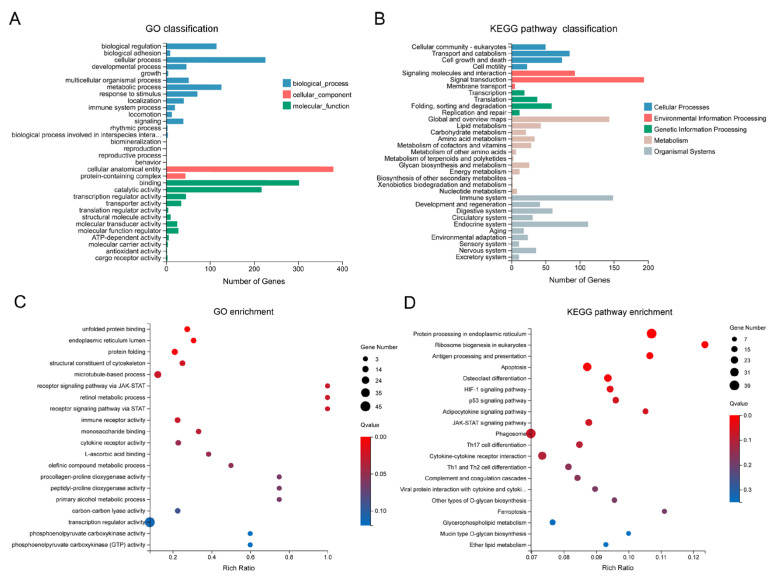
GO and KEGG analysis of the DEGs. (**A**) GO classification of the DEGs; (**B**) KEGG pathway classification of the DEGs; (**C**) GO enrichment analysis of the DEGs. The top 20 enriched GO terms are displayed, with dot size indicating the number of genes in each term and color representing the enrichment FDR (q-value); (**D**) KEGG pathway enrichment analysis of the DEGs. The top 20 enriched KEGG pathways are shown, with dot size indicating the number of genes in each pathway and color representing the enrichment FDR.

DEGs were categorized into three Gene Ontology (GO) terms, namely biological process (BP), cellular component (CC), and molecular function (MF), and further classified into five KEGG categories (Figure 3A,B). These DEGs primarily exhibited molecular functions of binding and catalytic activity and were involved in a series of biological processes, including metabolic processes and responses to stimuli (Figure 3A). Approximately 200 DEGs involved in signal transduction were assigned to the environmental information processing category (Figure 3B). Within the organismal systems categories, the top two were the immune system (149 genes) and the endocrine system (112 genes). GO enrichment analysis revealed that DEGs were significantly enriched (FDR < 0.05) in the top 10 terms, including unfolded protein binding, endoplasmic reticulum lumen, structural constituent of the cytoskeleton, and microtubule-based process (Figure 3C).

In total, DEGs were enriched in 243 KEGG pathways, with the top 6 significantly enriched (FDR < 0.05) pathways including protein processing in the endoplasmic reticulum, ribosome biogenesis in eukaryotes, antigen processing and presentation, and apoptosis (Figure 3D). To distinguish the pathways involved in up- and down-regulated genes, enrichment analyses were performed for these two gene sets separately. Up-regulated genes were significantly enriched (FDR < 0.05) in five pathways, including protein processing in the endoplasmic reticulum, the JAK-STAT signaling pathway, and two immune-related pathways—complement and coagulation cascades, and antigen processing and presentation (Supplementary Figure S2A). Down-regulated genes were significantly enriched (FDR < 0.05) in three pathways, namely gap junction, ribosome biogenesis in eukaryotes, and alpha-linolenic acid metabolism (Supplementary Figure S2B). Notably, up-regulated genes were also associated with ribosome biogenesis in eukaryotes, although this enrichment was not statistically significant (FDR > 0.05, Supplementary Figure S2A). These results collectively suggest that the ribosome biogenesis in eukaryotes pathway is profoundly affected by heat stress.

### 3.4. Immune- and Neuroendocrine-Related Pathways

**Figure 4:**
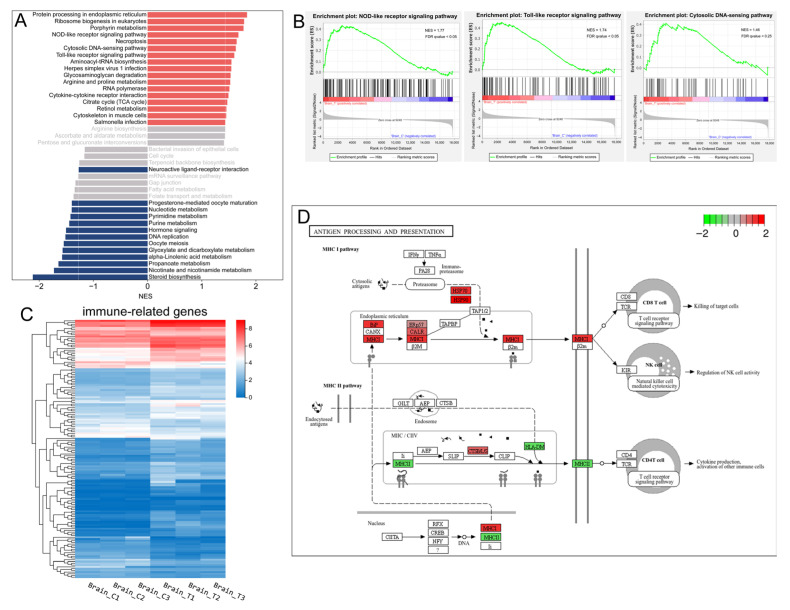
Immune-related genes and pathways. (**A**) the top 20 significantly up-regulated (red) and down-regulated (dark blue) gene sets identified by GSEA of the transcriptomic data based on KEGG pathway annotations (*p*-value < 0.05, FDR < 0.25). The normalized enrichment score (NES) for each pathway was calculated and is displayed in the histogram; (**B**) GSEA enrichment plots of the three up-regulated immune-related pathways; (**C**) Heatmap of the identified DEGs associated with the immune system. Colors represent the FPKM values of each gene; (**D**) The significantly enriched immune-related pathways in the context of DEGs. The color coding indicates the log_2_FC value of each gene, with red denoting up-regulated genes and green denoting down-regulated genes.

Neuroendocrine-immune system interactions are of physiological importance and are evolutionarily conserved. KEGG classification analysis indicated that the immune system and endocrine system were among the most prominently affected categories (Figure 3B). Furthermore, GSEA based on KEGG pathway annotations identified three immune-related pathways among the top 10 upregulated gene sets: the NOD-like receptor (NLR) signaling pathway, Toll-like receptor (TLR) signaling pathway, and cytosolic DNA-sensing pathway (Figure 4A,B, Supplementary Figure S3). The expression patterns of 149 immune system-associated DEGs are visually summarized in the heatmap presented in Figure 4C. KEGG enrichment analysis revealed that genes involved in the complement and coagulation cascades as well as antigen processing and presentation were up-regulated in the treatment group (Supplementary Figures S2A and S3). Taking the complement and coagulation cascades as an example, we observed the up-regulation of multiple complement components, including *C3* (*LOC119899479* and *LOC119918946*), *C4* (*LOC119897763* and *LOC119897764*), *C7* (*c7b*), and *C5AR1* (*c5ar1*), which suggests a state of chronic, low-grade inflammation mediated by the complement system. For antigen processing and presentation, all members of the MHC-I pathway—including *BiP* (*hspa5*), *HSP70* (*hspa8b*, *hspa4a*, *LOC119895688*, and *hspa1b*), *HSP90* (*hsp90aa1.1* and *hsp90aa1.2*), *MHC-I* (*LOC119907865*), and *CALR* (*LOC119901859* and *LOC119896814*)—were up-regulated, whereas the MHC-II pathway was down-regulated (Figure 4D). Collectively, these results suggest that long-term heat stress induces a state of neuroinflammation in the brain.

KEGG analysis found that the endocrine-related genes were primarily enriched in the adipocytokine signaling pathway (*p*-value = 0.0019, FDR = 0.058) (Figure 3D). In this pathway, the *leptin* (*LOC119893711*), *SOSC3* (*socs3a* and *socs3b*), *JAK* (*jak2b*), *STAT3* (*stat3*), *PEPCK* (*pck1* and *pck2*) were all up-regulated, while the leptin receptor (*lepr*) and *GLU1*(*slc2a1b*) were down-regulated. GSEA results demonstrated that the down-regulated gene sets were significantly enriched in two endocrine system-related pathways: hormone signaling and progesterone-mediated oocyte maturation. Additionally, steroid biosynthesis and neuroactive ligand–receptor interaction were also significantly inhibited (Figure 4A).

### 3.5. Cell Death Related Pathways

**Figure 5:**
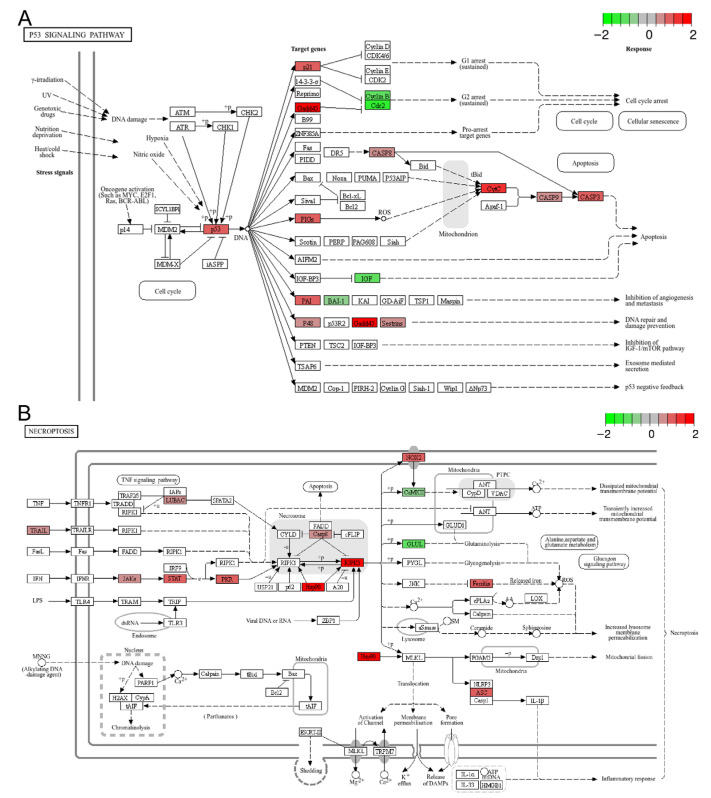
Representative enriched cell death related pathways: (**A**) the p53 signaling pathway; (**B**) necroptosis. The color scale represents the log_2_FC value for each gene, with red indicating up-regulation and green indicating down-regulation.

Numerous studies have demonstrated that both acute and chronic heat stress can induce cell death in various tissues [6,22,43]. In the present study, KEGG analysis identified three cell death-related pathways among the top 20 enriched pathways for DEGs and up-regulated genes, including apoptosis, the p53 signaling pathway, and ferroptosis (Figure 3D and Figure 5A and Figure S3). However, only apoptosis was significantly enriched (FDR < 0.05) in the context of DEGs (Figure 3D). Taking the well-known pro-apoptotic p53 signaling pathway as an example (Figure 5A), the up-regulation of *p53* (*tp53*), *p21* (*cdkn1a*), *CASP8* (*LOC119902446*), *CASP9* (*LOC119915381*), *CASP3* (*LOC119888653*), *CytC* (*LOC119904122*), together with the down-regulation of *IGF* (*igf1*), *Cyclin B* (*ccnb1*), and *Cdc2* (*cdk1*), indicated the activation of apoptotic cell death and the inhibition of cell proliferation in the treatment group. Additionally, GSEA of the transcriptomic data revealed that necroptosis was significantly up-regulated in the treatment group (*p*-value < 0.001, FDR < 0.05) (Figure 4A and Figure 5B). Collectively, these data suggest that chronic heat stress induces cell death and inhibits cell proliferation in the brain, albeit to a slight extent.

### 3.6. Heat Shock Protein Genes and Protein Synthesis and Processing

**Figure 6:**
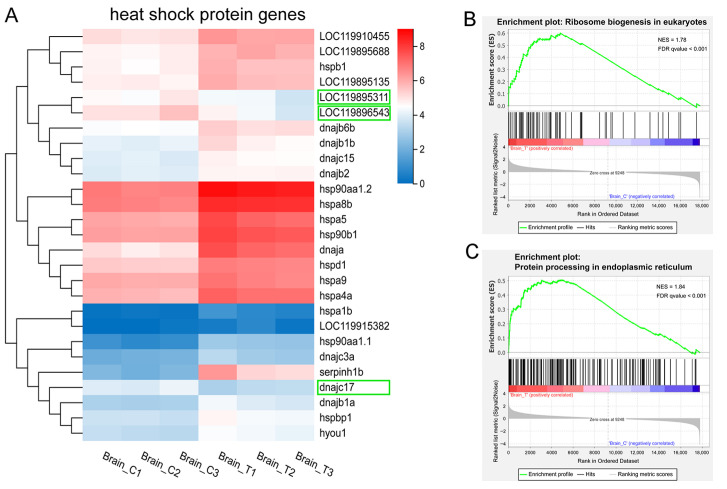
HSP genes and protein translation and post-translational processing. (**A**) heatmap of the DEGs encoding HSPs and their cochaperones. Colors indicate the FPKM values of each gene. Downregulated genes are highlighted in green boxes; (**B**–**C**) GSEA enrichment plots of (**B**) ribosome biogenesis in eukaryotes and (**C**) protein processing in endoplasmic reticulum pathways.

HSPs are molecular chaperones that play a crucial role in stress resistance and environmental adaptation by facilitating the folding of newly synthesized polypeptides. They are classified into five families based on their molecular weights: HSP90, HSP70, HSP60, HSP40, and small heat shock proteins (sHSPs). Under normal conditions (control group), some HSP genes were expressed at relatively high levels. Specifically, *hsp90aa1.2* and *hspa8b* showed the highest expression (FPKM > 100). Genes with low expression included *LOC119915382*, *hsp90aa1.1*, *serpinh1b*, *hspa1b*, and *dnajc3a* (FPKM < 5, Figure 6A).

In this study, we identified 27 DEGs that are HSPs or their co-chaperones (Figure 6A). Among these, 22 HSP genes and 2 cochaperones were significantly up-regulated in the treatment groups (Figure 6A), including members of the HSP90 family (*hsp90aa1.1*, *hsp90aa1.2*, and *hsp90b1*), HSP70 family (*hspa1b*, *hspa8b*, *hspa5*, *hspa4a*, *LOC119895688*, *hspa9*, and *hyou1*), HSP60 family (*hspd1*), HSP40 family (*serpinh1b*, *dnaja*, *LOC119915382*, *dnajc3a*, *dnajb1a*, *dnajb1b*, *dnajb2*, *dnajb6b*, and *dnajc15*), and sHSP family (*hspb1* and *LOC119895135*). Notably, *serpinh1b* (*hsp47*), a member of the serpin superfamily, showed the most significant up-regulation (FC = 11.49) in high-temperature groups. Additionally, *LOC119910455* and *hspbp1*—cochaperones of HSP90 and HSP70, respectively—were also significantly up-regulated. In contrast, two HSP70 family genes (*LOC119895311* and *LOC119896543*) and one HSP40 family gene (*dnajc17*) were significantly down-regulated in the treatment groups, though the underlying mechanism remains unclear.

The up-regulation of HSP genes was consistent with the GSEA results showing up-regulated ribosome biogenesis in eukaryotes and protein processing in the endoplasmic reticulum (Figure 4A and Figure 6B,C). Collectively, these findings demonstrate the activation of a robust protein quality control system that functions to refold, stabilize, or degrade heat-damaged proteins, thereby mitigating cellular proteotoxicity under thermal stress. The concurrent enrichment of the “ribosome biogenesis” pathway should not be interpreted as enhanced global translation—a process typically suppressed under severe heat shock. Instead, it likely reflects a preparatory strategy to facilitate rapid cellular recovery and resumption of protein synthesis once the stress conditions alleviate.

### 3.7. Microtubule-Based Process and Cell Division

**Figure 7:**
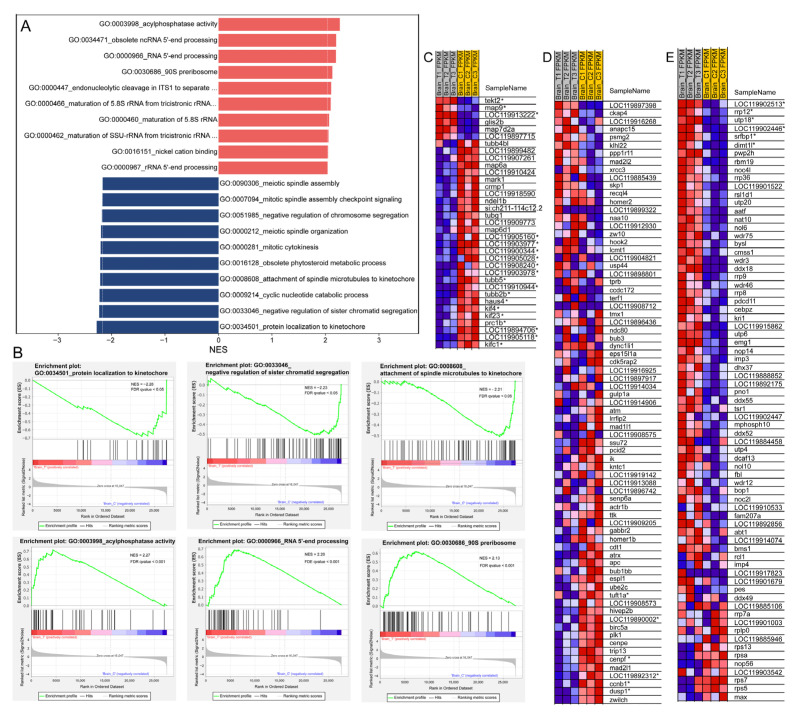
GSEA of transcriptomic data based on GO annotations. (**A**) Histogram showing the top 10 up-regulated (red) and down-regulated (dark blue) gene sets with their corresponding normalized enrichment scores (NES); (**B**) Representative GSEA enrichment plots of down-regulated (upper panel) and up-regulated (lower panel) gene sets; (**C**–**E**) Heatmaps of selected GSEA results: (**C**) “microtubule-based process” (GO:0007017); (**D**) “negative regulation of sister chromatid segregation” (GO:0033046); (**E**) “90S preribosome” (GO:0030686). In the heatmaps and enrichment plots, red indicates a strong positive association between gene expression levels and the treatment, whereas blue indicates a negative correlation between gene expression levels and the treatment. DEGs in the heatmaps are marked with an asterisk (*).

GO enrichment revealed that the DEGs were significantly enriched in “microtubule-based process” and “structural constituent of cytoskeleton” (Figure 3C). Moreover, GSEA based on GO annotations showed that down-regulated genes were mainly enriched in mitosis-related gene sets, including “protein localization to kinetochore” (GO:0034501), “negative regulation of sister chromatid segregation” (GO:0033046), “attachment of spindle microtubules to kinetochore” (GO:0008608), and “mitotic cytokinesis” (GO:0000281) (Figure 7A,B,D). Additionally, cell division is closely associated with microtubule-based processes; in this context, cytoskeleton-associated tubulin genes (*LOC119905160*, *LOC119903977*, *LOC119900344*, *LOC119905028*, *LOC119903978*, *LOC119905118*, *tubb5*, and *tubb2b*) and kinesin genes (*kif4*, *kif23*, *kifc1*, and *LOC119894706*) were all significantly down-regulated (Figure 7C). Besides, the DNA replication and cell cycle were also inhibited according the GSEA results (Figure 4A). In contrast, up-regulated genes were enriched in ribosome biogenesis-related gene sets, such as the 90S preribosome (GO:0030686, Figure 7B,E), which was consistent with the results of GSEA based on the KEGG pathway annotations (Figure 3D and Figure 4A).

### 3.8. PPI Analysis

**Figure 8:**
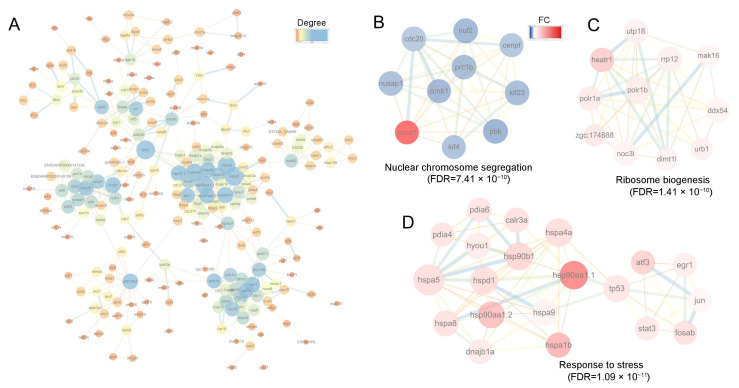
PPI and clustering analysis of the DEGs. (**A**) PPI analysis of DEGs, where node color indicates the degree of connectivity (1–25 connections). Node size also corresponds to the degree; (**B**–**D**) The top 3 clusters of DEGs analyzed using MCODE plugin in Cytoscape software. In (**B**–**D**) node color represents the FC of each gene, with the range from 0 to 7; red denotes upregulated genes, and blue denotes downregulated genes. For the network edges in (**A**–**D**), their color and thickness reflect the interaction confidence, with a minimum threshold set at 0.70.

To further investigate the relationships among the DEGs, we conducted a PPI analysis. This revealed complex interaction networks (Figure 8A). Cluster analysis of the sub-networks using the MCODE algorithm identified 41 key genes in the top three clusters (Figure 8B–D). These results were generally consistent with those obtained via the topological analysis method MCC (Supplementary Table S4). The hub genes were involved in three biological processes: nuclear chromosome segregation (FDR = 7.41 × 10^−10^), ribosome biogenesis (FDR = 1.41 × 10^−10^), and response to stress (FDR = 1.09 × 10^−11^) (Figure 8B–D). The core genes associated with chromosome segregation included *cenpf*, *nuf2*, *nusap1*, *kif23*, *kif4*, *ccnb1*, and *prc1b*. For ribosome biogenesis, the core genes were *dimt1l*, *heatr1*, *urb1*, *ddx54*, *mak16*, *utp18*, *rrp12*, and *zgc:174888*. The core genes involved in the cellular response to stress included *hspa8*, *hspa1b*, *hspd1*, *hsp90b1*, *hspa9*, *hsp90aa1.1*, *hsp90aa1.2*, *hyou1*, *pdia4*, *calr3a*, *tp53*, *atf3*, and *jun*. Notably, all genes involved in chromosome segregation were down-regulated except for *ccna1*. In contrast, genes involved in ribosome biogenesis and stress response were significantly up-regulated (Figure 8B–D).

## 4. Discussion

Global warming has exerted adverse impacts on aquaculture; however, the mechanisms underlying teleost fish’s responses to temperature changes remain incompletely understood. This study is expected to provide crucial insights into the molecular processes associated with the effects of high temperatures on the fish brain—a central and temperature-sensitive organ. In contrast to the systemic immunosuppression observed in peripheral organs such as the spleen and liver, the brain exhibits distinct immunomodulatory adaptations. Concomitant with the cytoarchitectural changes observed in the brain following prolonged exposure to high temperatures, significant molecular alterations were detected, including the up-regulation of *olig2*. As a well-recognized marker of oligodendrocytes—cells critical for maintaining CNS homeostasis [44]—the up-regulation of *olig2* suggests the plasticity of neural circuits, which may enable fish to adapt to changing environmental conditions. These molecular-level changes reflect the acclimatization mechanisms that support fish survival under high-temperature stress.

### 4.1. Heat Stress Induces Heat Shock Protein Genes

Heat stress elicits significant molecular responses in fish, particularly via the upregulation of HSPs—molecules that play a central role in cellular protection and the maintenance of homeostasis [45]. HSPs facilitate the folding of newly synthesized polypeptides, prevent protein aggregation by refolding or degrading misfolded proteins, and preserve proteome integrity under stress conditions. The HSP70 and HSP90 families are among the primary mediators of these functions [45]. In the LMB brain, we observed pronounced expression of key HSP genes, among which *hsp90aa1.2* (HSP90 family) and *hspa8b* (HSP70 family) were the most abundant. Notably, *serpinh1b* and *hsp90aa1.1*, though expressed at low levels in the control group, exhibited dramatic increases under heat stress (>10-fold and >5-fold, respectively). This suggests their high thermal sensitivity and potential utility as biomarkers for thermal stress, which aligns with findings in other fish species [46,47,48].

Additionally, *Hsp40* (*DnaJ*) emerged as particularly noteworthy, accounting for approximately 40% (9/22) of the upregulated HSP genes in the LMB brain. As co-chaperones of HSP70, Hsp40 proteins not only support protein quality control but also contribute to immune regulation [49]. Beyond their chaperone roles, HSPs are integral to stress adaptation, as they modulate apoptosis, immune responses, and inflammation. Their elevated expression under thermal (or other) stressors enhances cellular resilience, enabling survival despite adverse conditions [45]. The upregulation of HSP genes observed in this study is consistent with reported changes in the brains of various fish species following both acute and chronic thermal stress [1,5,33].

### 4.2. Heat Stress Induces Neuroinflammatory Signaling and Dysregulates Neuroendocrine Pathways in the Brain

Neuroimmune regulation is essential for the normal development and maintenance of animal health. The cytoarchitectural alterations in the PGZ of the optic tectum—characterized by a reduction in undifferentiated radial glial cells and an increase in neurofilaments—may lead to changes in neuronal activity. Notably, the neuroactive ligand–receptor interaction pathway, which was altered in our study, has also been reported to be disrupted in the brain of Chinese tongue sole following acute heat stress [16]. These changes in CNS architecture might be closely associated with neuroimmune adaptation. Both KEGG enrichment analysis based on DEGs and GSEA considering all transcriptomic data revealed the up-regulation of multiple immune-related pathways, indicating that long-term heat stress activates the immune system.

The NLR signaling pathway is a vital component of the innate immune system, enhancing immune, apoptotic, and inflammatory responses to support cellular survival [50]. High temperatures may indirectly affect JAK/STAT signaling pathways via HSPs, thereby upregulating genes associated with NLR signaling [50]. NLR family proteins mediate the initial innate immune response to cellular injury and stress [51]. For instance, acute thermal stress activates the NLR signaling pathway in the liver of black rockfish [52]. Consistently, our study observed concurrent activation of NLR signaling through both inflammasome-dependent (NLRP1-ASC) and -independent (NOD1-NF-κB/MAPK) pathways, indicating a robust, sustained innate immune response to chronic thermal challenge [51]. Additionally, activation of another pattern recognition receptor pathway—TLR signaling pathway—was detected; this pathway produces cytokines and numerous inflammatory mediators upon recognizing pathogen-associated molecular patterns (PAMPs) [53], further supporting the induction of a chronic neuroinflammatory state in the LMB brain under chronic heat stress.

Adaptive immune responses involve antigen processing and presentation pathways, which present peptide antigens to CD8^+^ and CD4^+^ T cells via MHC class I and II molecules, respectively [54]. In the LMB brain, the adaptive immune system exhibited selective modulation: genes in the MHC I pathway were upregulated to enhance endogenous antigen presentation, while MHC II was downregulated—likely a protective mechanism to prevent excessive neuroinflammation [54]. In stark contrast, genes of the MHC class I pathway were unexpectedly upregulated. While this enhances the machinery for endogenous antigen presentation, its role during a sterile, chronic stressor is likely maladaptive. In the absence of a pathogen, the persistent upregulation of MHC I on neural cells can act as a stress signal, potentially increasing their vulnerability to immune-mediated damage and contributing to synaptic dysfunction. This brain-specific upregulation of MHC I is particularly noteworthy as it directly opposes the response in peripheral tissues, where MHC I is typically suppressed as part of a systemic immunosuppressive state during heat stress [55].

Our study further reveals that chronic heat stress triggers complex neuroendocrine-immune crosstalk in the fish brain, mediated through both the HPI axis and metabolic signaling pathways [6,56]. Notably, we observed dysregulation of the adipocytokine signaling pathway: upregulated *leptin* expression may serve dual roles, suppressing appetite through central mechanisms and acting as a pro-inflammatory cytokine that likely exacerbates the ongoing neuroinflammation [57,58]. However, concurrent upregulation of *socs3* and downregulation of *lepr* suggest strong indicators of central leptin resistance. This pathological state of impaired metabolic signaling may lead to a misallocation of energy resources, which can then inadvertently fuel the detrimental inflammatory processes observed in the brain [57]. This metabolic-immune interplay is coordinated through the interleukin-6 (IL-6)-initiated JAK2-STAT3 pathway. This pathway regulates energy homeostasis by suppressing melanocortin production and also exerts specific functions in immune regulation [57,59,60]. Notably, this pathway was also affected by acute heat stress in the brain of Chinese tongue sole [16], indicating an intrinsic connection within the neuroendocrine system.

Collectively, these findings suggest that LMB survival under chronic heat stress relies on a sophisticated neuroendocrine-immune rebalancing in the brain, which maintains protective immunity while preventing excessive inflammation. The observed complement activation may facilitate damage control and synaptic maintenance, while NLR and TLR pathway activation provides continuous stress surveillance. This multi-layered neuroimmune response represents a strategy to preserve critical neural functions under prolonged thermal stress [56].

### 4.3. Heat Stress Induces Cell Death and Inhibits Cell Proliferation

Cells possess various protective mechanisms against stress; however, excessive stress can overwhelm these defenses, leading to disrupted signaling, DNA damage, and cell death [61]. Heat stress, in particular, can induce cell death in multiple tissues of fish [1,6,22,23,24,34,35,43]. In our study, DEGs were enriched in three key cell death pathways: apoptosis, the p53 signaling pathway, and ferroptosis. Apoptosis, a well-characterized form of programmed cell death, plays a crucial role in development, cellular homeostasis, and immune responses [62]. It can be triggered via extrinsic and intrinsic pathways: the intrinsic pathway is activated by cellular perturbations such as DNA damage, withdrawal of growth factors (e.g., down-regulated *igf1*), and mitochondrial impairment; the extrinsic pathway is initiated by death receptor ligands (e.g., TRAIL), leading to the activation of caspase-8/9 and the release of mitochondrial cytochrome c, which ultimately activates caspase-3 [62,63,64]. Additionally, p53 activation (*tp53* up-regulation) and its downstream target *p21* may induce cell cycle arrest at G2/M by suppressing *ccnb1* and *cdk1* [65].

Microtubules, which provide structural support in eukaryotic cells, are critical for diverse cellular processes including chromosome segregation during mitosis [66]. They also facilitate the dynamic redistribution of organelles and adapt to internal and external stimuli by rearranging the cytoskeleton [67,68]. Our findings demonstrate that chronic heat stress induces significant disruptions to both microtubule dynamics and chromosomal stability mechanisms in LMB brains. The coordinated down-regulation of microtubule-associated genes and key components of chromosome segregation suggests a systemic impairment of cellular architecture and division fidelity. This dual disruption may lead to severe chromosomal instability (CIN), triggering cell cycle arrest and death [69]. The hub genes identified via PPI analysis were involved in chromosome segregation, underscoring their critical role in maintaining cellular homeostasis. The reduction in undifferentiated radial glial cells and increase in nerve fibers might result from the inhibition of cell proliferation, which could promote glial cell differentiation. Notably, acute heat stress has been shown to induce expression changes in cytoskeleton organization-related proteins in the zebrafish brain [5], suggesting a universal impact of thermal perturbations on cellular microstructure. These findings provide important mechanistic insights into neural vulnerability to climate change, revealing how sustained thermal stress can simultaneously compromise both structural (microtubule-based) and functional (chromosomal segregation) cellular integrity.

### 4.4. Altered Regulation of Ribosome Biogenesis Under Heat Stress

Ribosome biogenesis is an energy-intensive process that is crucial for cellular homeostasis, growth, and stress responses [70,71,72]. Previous studies have reported varied ribosomal responses to thermal stress: for instance, it is upregulated in cold-stressed zebrafish [73] but suppressed in heat-acclimated *Clarias fuscus* [7]. In contrast, our PPI analysis revealed consistent upregulation of ribosome biogenesis-related genes (e.g., *dimt1l*, *heatr1*, *urb1*) in the LMB brain. The induction of key genes like *heatr1* is particularly significant. Given that its disruption is known to trigger p53-mediated cell cycle arrest [71,74], its sustained upregulation suggests a defensive response to protect the integrity of the ribosome assembly process, thereby staving off a p53-triggered cellular shutdown. This highlights that ribosome biogenesis is under severe strain, rather than being smoothly ‘enhanced’.

The consistent upregulation of these genes presents a biological paradox, given that global protein translation is typically suppressed during severe heat stress to conserve energy and prevent proteotoxicity. Therefore, this response is unlikely to support a general enhancement of protein synthesis during the stress period. Instead, we propose this reflects a critical compensatory or preparatory strategy. The sustained upregulation may be necessary to counteract stress-induced damage to ribosomal components and maintain a minimal functional pool. Alternatively, it could be a preparatory mechanism to ensure a rapid resumption of protein synthesis and cellular recovery after the stressor is removed. Collectively, our findings identify ribosome biogenesis, together with stress response gene sets, as key regulatory nodes in thermal adaptation, with tissue-specific responses reflecting distinct strategies for energy allocation.

## 5. Conclusions

In conclusion, we have uncovered a novel neuroimmune regulatory and adaptive mechanism in the brain of LMB under chronic heat stress, which is characterized by three key features: (1) activation of immune pathways, (2) sustained enhancement of heat stress responses, and (3) compensatory upregulation of ribosome biogenesis. This coordinated response balances cell death processes with immune defense mechanisms, thereby enabling survival under elevated temperatures. These findings underscore the complex trade-offs inherent in thermal adaptation. Future studies should investigate how stress intensity and duration modulate these responses, which will contribute to a better prediction of climate change impacts on aquatic species.

## Data Availability

Raw RNA-seq data are publicly available and have been deposited in the Genome Sequence Archive (GSA) at the China National Center for Bioinformation (CNCB) under accession number CRA027785. The data can be accessed at https://ngdc.cncb.ac.cn/gsa/.

## Supplementary Materials

The following supporting information can be downloaded at: https://www.mdpi.com/article/10.3390/ani15213067/s1, Figure S1: Correlation of relative gene expression levels estimated by RNA-Seq and qPCR; Figure S2: KEGG pathway enrichment analysis of the up- and down-regulated DEGs; Figure S3: Gene expression in the immune-related pathways; Table S1: Primers for qPCR analysis of the selected differentially expressed genes; Table S2: Quality control and preprocessing of the sequencing data; Table S3: Differentially expressed genes in the brains of largemouth bass from control versus treatment groups. Table S4: Hub genes of the PPI network analyzed with MCC and MCODE.

## Abbreviations

The following abbreviations are used in this manuscript:

LMB	Largemouth bass
DEGs	Differentially expressed genes
KEGG	Kyoto Encyclopedia of Genes and Genomes
GO	Gene Ontology
GSEA	Gene set enrichment analysis
PPI	Protein–protein interaction
HPI	Hypothalamic–pituitary–interrenal
HSP	Heat shock protein
PGZ	Periventricular gray zone
PCA	Principal Component Analysis
MCC	Maximal Clique Centrality
NLR	NOD-like receptor
TLR	Toll-like receptor
FC	Fold change
FPKM	Fragments per Kilobase of Transcript per Million fragments mapped
CNS	Central nervous system
