# Supplementary material for: Long-Term Heat Stress Triggers Immune Activation and Cell Death Remodeling in the Brain of Largemouth Bass (Micropterus salmoides)
Source: Animals (Basel). 2025 Oct 22;15(21):3067. doi: 10.3390/ani15213067 (PMC12610153; doi:10.3390/ani15213067)

Supplementary Figure S1. Correlation of relative gene expression levels estimated by RNA-Seq and qPCR. **(A)** Histogram comparing the relative gene expression levels between RNA-Seq and qPCR based on log2 normalized fold changes. **(B)** Consistency between DEGs identified via RNA-Seq analysis and qPCR. The x-axis and y-axis represent relative expression values from RNA-Seq and qPCR, respectively. The R value denotes the Pearson correlation coefficient. All figures were plotted using GraphPad software (version 9.0.0).

Supplementary Figure S2. KEGG pathway enrichment analysis of the up- and down-regulated DEGs. **(A)** the top 20 enriched KEGG pathways of the up-regulated DEGs. (B) the top 20 enriched KEGG pathways of the down-regulated DEGs.

Supplementary Figure S3. Gene expression in the immune-related pathways. **(A)** NOD-like receptor signaling pathway, **(B)** Toll-like receptor signaling pathway, and **(C)** complement and coagulation cascades were plotted with DEGs by PATHVIEW (Luo *et al.*, Bioinformatics, 2013). Red represents up-regulated genes, while green represents down-regulated genes in the treatment group.

Supplementary Figure S1

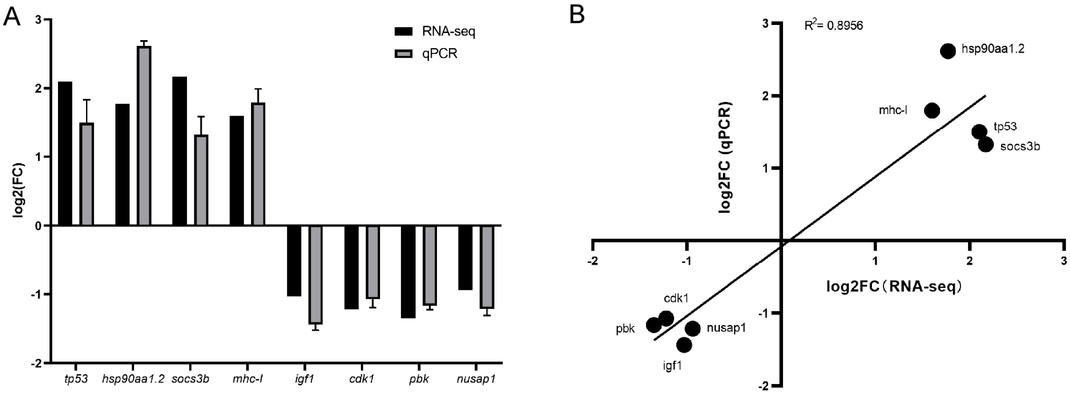

Supplementary Figure S2

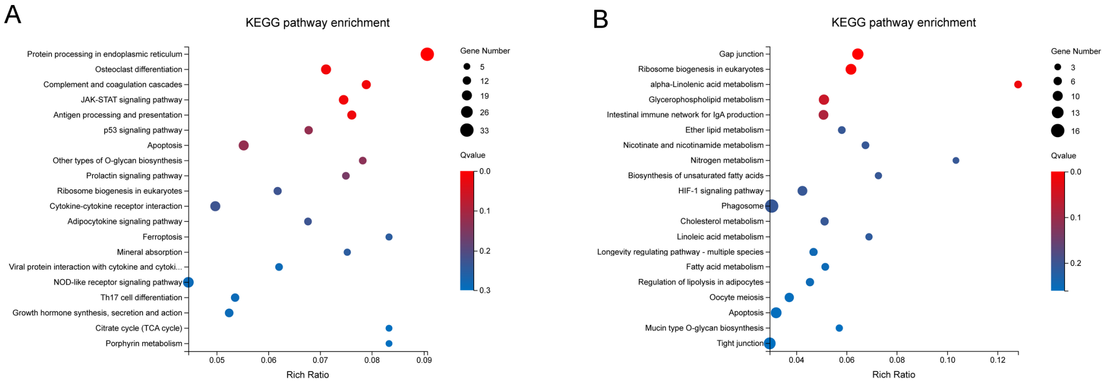

Supplementary Figure S3

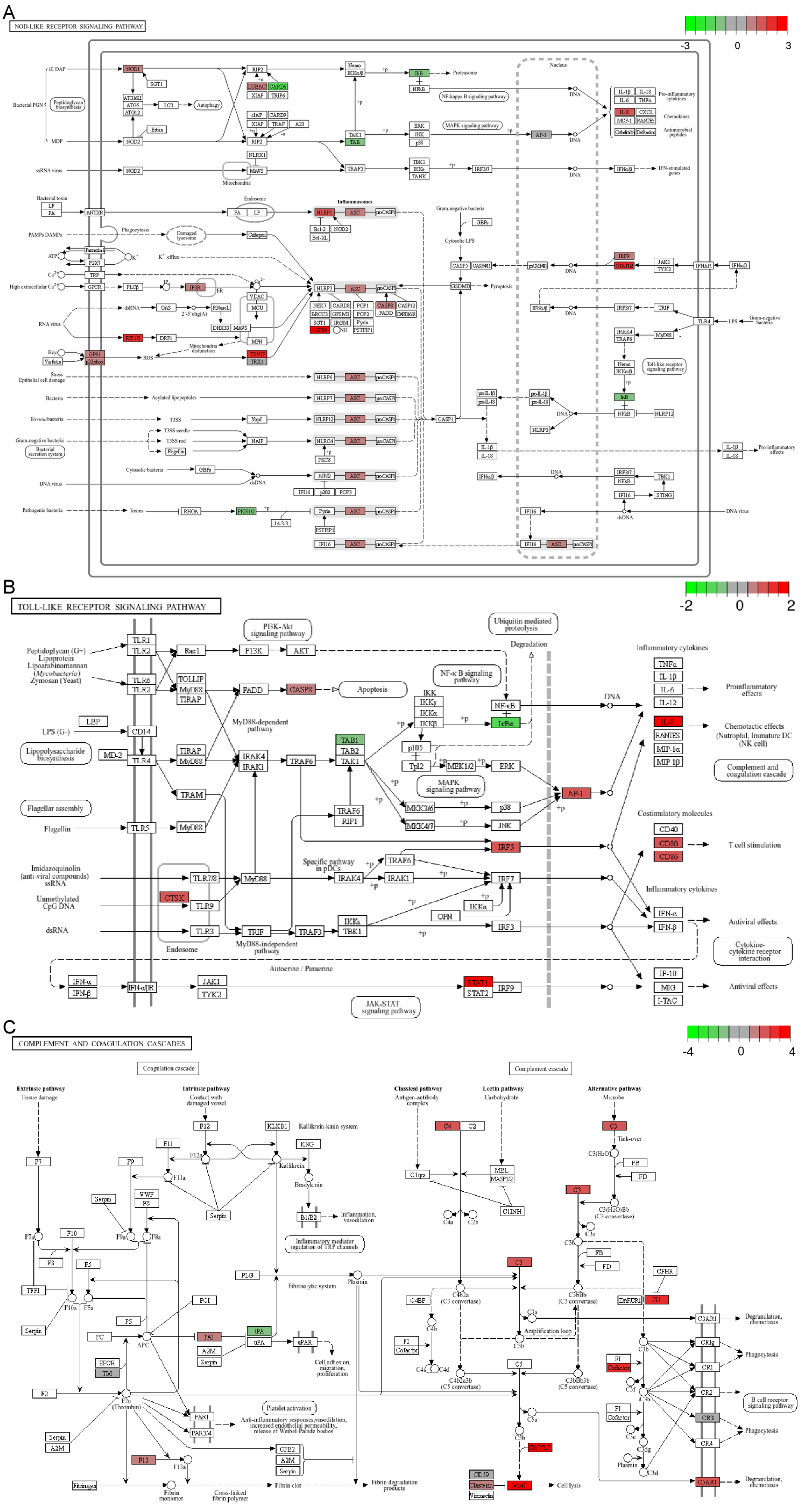

Supplement: Supplementary file 1 [file animals-15-03067-s001.zip › Supplementary Figures.pdf]
